# Analysis of post-market adverse events of tafamidis base on the FDA adverse event reporting system

**DOI:** 10.1038/s41598-024-64697-y

**Published:** 2024-06-13

**Authors:** Fan Wu, He Zhu, Yue Zhang

**Affiliations:** 1 Department of Pharmacy, Fuwai Yunnan Hospital, Chinese Academy of Medical Sciences, Kunming, China; 2 Pharmacy Department, Yan’an Hospital of Kunming City, Kunming, China

**Keywords:** Tafamidis, Amyloid cardiomyopathy, Adverse event, Real-world data-mining, Pharmacovigilance, Drug safety, Pharmacology, Clinical pharmacology

## Abstract

Tafamidis is the world's first and only oral drug approved to treat the rare disease transthyretin amyloid cardiomyopathy (ATTR-CM). Medicines are known to have different adverse reactions during the course of treatment. However, the current limited clinical studies did not identify significant adverse drug reactions to tafamidis. Tafamidis has been on the market for 5 years now, a large number of adverse drug event (ADE) reports with tafamidis as the primary suspected drug have been reported in the United Food and Drug Administration's adverse event reporting system (FAERS). We retrieved 8170 adverse event reports in FAERS with tafamidis as the first suspected drug, and mined these reports for positive signals to perform risk warnings for potentially possible adverse events with tafamidis. We found that a large number of adverse events associated with the primary disease were reported due to insufficient awareness of ATTR among the reporters, leading to a large number of positive signals reported in the cardiac disorders system. We also found that tafamidis has the potential to cause an adverse event risks of ear and labyrinth disorders system and urinary tract infection bacterial, which deserve continued clinical attention.

## Introduction

Transthyretin (TTR) is a transport protein in the blood that is responsible for transporting thyroxine and retinol through the body. TTRs are usually present as homotetrameric proteins that are stable. When the trans-TTR gene is mutated, the TTR protein tetramer structure becomes less stable and tends to dissociate into monomers, misfit, and aggregate, forming amyloid fibril aggregates. A mutation in the gene encoding TTR results in the TTR protein tetramer structure becoming less stable and tending to dissociate into monomers, misfit, and aggregate, resulting in tissue deposition of the protein as insoluble amyloid fibrils. The deposition of insoluble amyloid fibrils in the nervous system is called familial amyloid polyneuropathy (FAP), which manifests as neuropathic pain, sensory abnormalities, impaired motor function, muscle weakness, and an inability to walk. The deposition of insoluble amyloid fibrils in the interstitium of the cardiac muscle is called transthyretin amyloid cardiomyopathy (ATTR-CA), which manifests as myocardial pathology, cardiac arrhythmias, and heart failure. If patients do not receive therapeutic interventions, progressive disability may occur, patients may have a poor quality of life, and ultimately end up dying^1^. Relevant reports indicate that the median survival of wild-type ATTR-CA patients after diagnosis is only 43–57 months^2,3^. Patients with the Val122ILe mutant phenotype typically survive for only 31 months after diagnosis^3^. The FDA approved tafamidis in October 2019 for the treatment of ATTR-CA, making it the first and only drug approved for the treatment of ATTR-CM in the world. Tafamidis is a TTR kinetic-stabilizing agent, prevents the formation of insoluble amyloid fibrils by selectively binding to the thyroid hormone T4 receptor on the surface of the TTR, stabilizing the TTR protein tetramer and slowing down the dissociation of the TTR^4^. Clinical research has proven that tafamidis enhances the prognosis of patients with ATTR-CA. Compared to placebo tafamidis significantly reduced all-cause mortality and cardiovascular-related hospitalizations, slowed the rate of decline in quality of life and peripheral neurological impairment in patients with ATTR-CA, while being well tolerated^5^. Due to the advancements in diagnostic and treatment technologies, more and more hereditary transthyretin (ATTRv) amyloidosis patients are being diagnosed, which makes tafamidis' safety in clinical application a concern. Our study was based on the FDA adverse event reporting system (FAERS) for adverse reaction data mining, with a view to discovering potential adverse reactions during real-world application of tafamidis, and identifying new or rare adverse reactions to inform the safe clinical application of the drug^6,7^.

## Methods

### Data sources and processing

We gathered all reports of adverse events (AE) from the FAERS database that occurred between inception through the fourth quarter of 2023. Then we removed duplicate reports. ALL reports were classified and described using the system organ class (SOC) and preferred term (PT) using the medical dictionary for drug regulatory activities (MedDRA) (version 27.0)^8^. The AE category in the report pairs with the SOC and the AE name pairs with the PT. All raw data were recorded in Microsoft Excel, data processing and signal calculations were completed using R 4.3.2, statistical analyses were completed using SPSS 26.0, and all graphs were plotted using GraphPad Prism 9.5.1.

### Data extraction and descriptive analysis

We found adverse event reports between the fourth quarter of 2019 and the fourth quarter of 2023 and then screened all adverse event reports with "VYNDAMAX", "VYNDAQEL", "TAFAMIDIS" as the first suspected drugs. Based on these reports, we conducted data extraction and descriptive analysis, including basic information on cases and the timing and number of AE report.

### Signal detection and analysis

**Figure 1:**
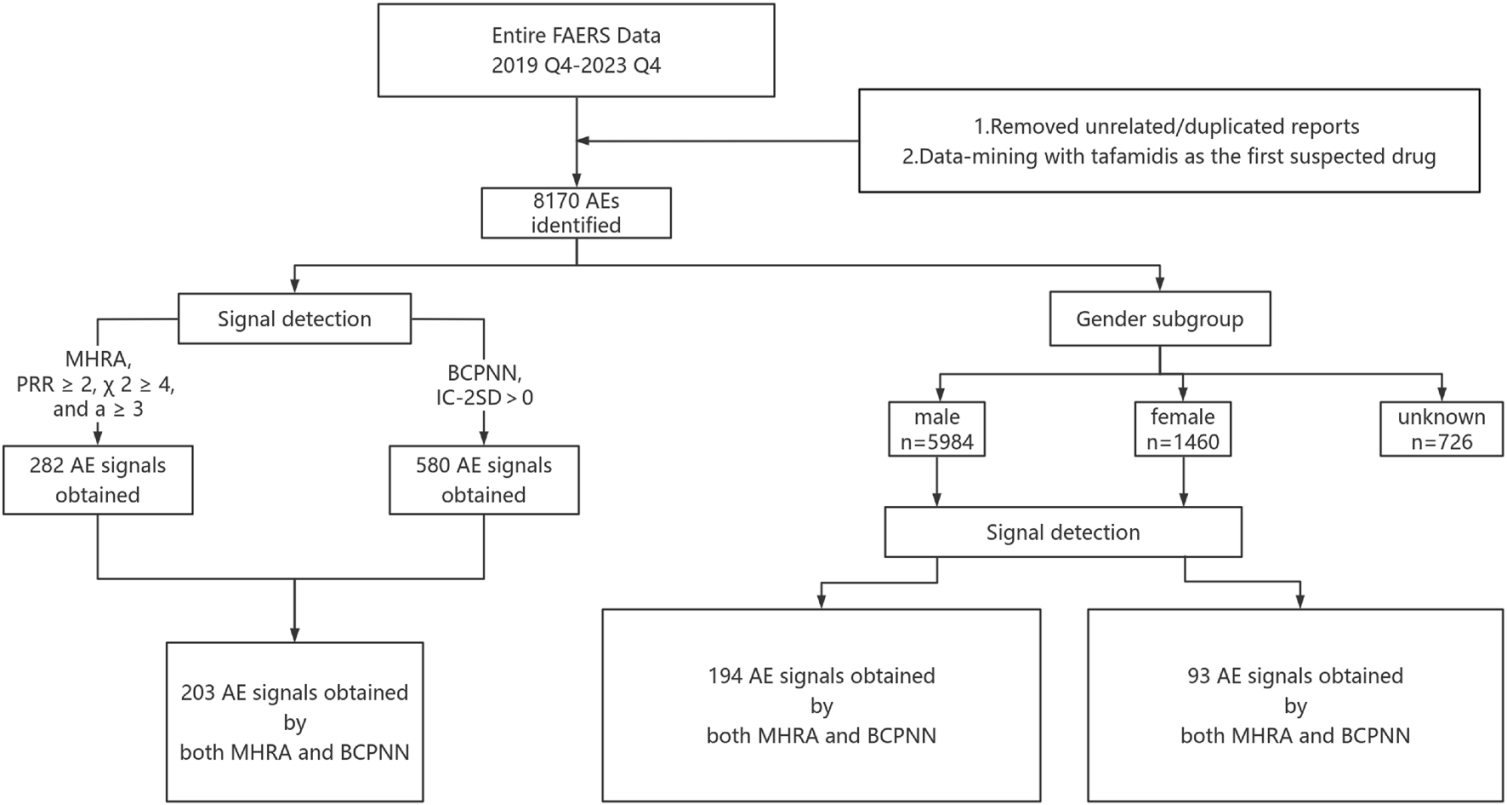
Flow chart of the data analysis.

The disproportionality analysis is the only method used for mining drug ADR risk in adverse event databases. Commonly used measures of disproportionality analysis include the Proportional Reporting Ratio (PRR), Reporting Odds Ratio (ROR), Medicines and Healthcare Products Regulatory Agency (MHRA), Bayesian Discriminant Confidence Interval Progressive Neural Network Modeling (BCPNN), and Relative Ratio (RR) methods. Regulatory Agency (MHRA), Bayesian Criterion Confidence Interval Progressive Neural Network (BCPNN), and Relative Ratio (RR)^6,9–11^. In this study, MHRA and BCPNN were used together for the positive signals screening to ensure the credibility and sensitivity of the mining results. The MHRA and BCPNN were calculated based on a four-cell table. In Equation, a means the number of reports containing both the target drug and target AE/SOC; b means number of reports containing other AE/SOC of the target drug; c means the number of reports containing the target AE/SOC of other drugs; d means the number of reports containing other drugs and other AE/SOC. Based on the MHRA and BCPNN formulas to perform the calculations (see supplementary material S1 for specific formulas and analytical methods). When the calculation result is satisfied with both a ≥ 3, χ^2^ ≥ 4, proportional reporting ratio (PRR) ≥ 2, lower limit of the information component (IC-2SD) > 0, one positive signal is recognized. A positive signal indicates that the target drug has a significant risk of causing this AE/SOC. Because of the serious data loss of patients' age in the AE reports, only the gender subgroup was analyzed in this study. The detailed screening process is shown in Fig. 1. Based on the positive signal results, we proceeded to use the chi-square test to compare the significance of the difference between the positive signal of the target drug and those of the other drugs. *P* < 0.05 indicates a significant difference. In addition, the reporting odds ratio confidence interval (ROR 95% CI) of the signals was calculated. If the 95% CI of the two signals do not overlap, then according to the principle of hypothesis testing, we can assume that there is a significant difference between the two signals. A forest plot is used to show the comparison of signal intensities.

### AE induce time analysis

There were some missing data in the FAERS database for the time of medication and time of AE, so we deleted reports missing either of these first and then calculated the induced time of the AE in each case report. Calculation formula: AE induce time = time of AE reporting − time of medication. Classified and counted the AE induce time by cardiac amyloidosis related, cardiac disorders, ear and labyrinth disorders, male, and female. We plotted the survival curves of different categories of AE induce time and analyzed the different categories of AE induce time using nonparametric tests, and *P* < 0.05 indicated that the difference was statistically significant.

## Results

### Basic information

We extracted 8170 validated AE reports with tafamidis as the first suspected drug from the FAERS database, as shown in Table 1. The rate of reported adverse events was significantly higher in male patients (73.24%) than in female patients (17.87%). No reports of minors and the large majority of the reports were older than 65 years old (80%). The country with the most reports was the United States (73.05%), followed by Japan (6.81%). The reported statistics of patients' final treatment outcomes mainly included death (28.46%), and hospitalization (17.43%).

**Table 1:** Basic information of tafamidis AE reports.

Item	Category	Case number	Case proportion (%)
Sex	Male	5984	73.24
	Female	1460	17.67
	Unknown	726	8.89
Age (years)	18–64	261	3.19
	65–85	4845	59.30
	> 85	1691	20.70
	Unknown	1373	16.81
Weight (kg)	< 50	115	1.41
	50–100 kg	2188	26.78
	> 100 kg	235	2.88
	Unknown	5632	68.94
Serious outcome	Hospitalization	1723	17.43
	Death	2813	28.46
	Life-threatening	97	0.98
	Disability	56	0.57
	Other	5195	52.56
Report recorder	Medical doctor	1785	21.85
	Physiotherapists	387	4.74
	Consumers	4355	53.30
	Other	1643	20.11
Reporter country	United States of America	5968	73.05
	Japan	556	6.81
	French	493	6.03
	Canada	373	4.57
	Other	780	9.55

### Timing and the number of AE reports

**Figure 2:**
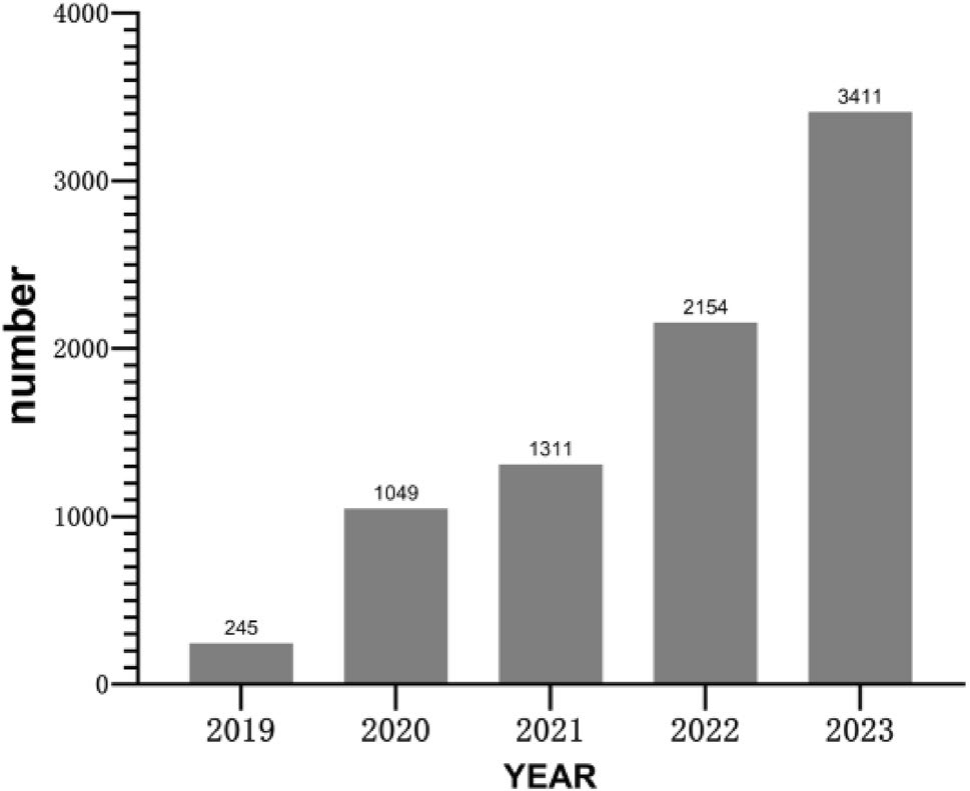
Timing and the number of AE report.

The year and number of AE reports are shown in Fig. 2. The number of tafamidis adverse event reports in FAERS has increased each year. 2023 was the most reported with n = 3411.

### Result of positive signal mining

#### Positive SOC signals

**Figure 3:**
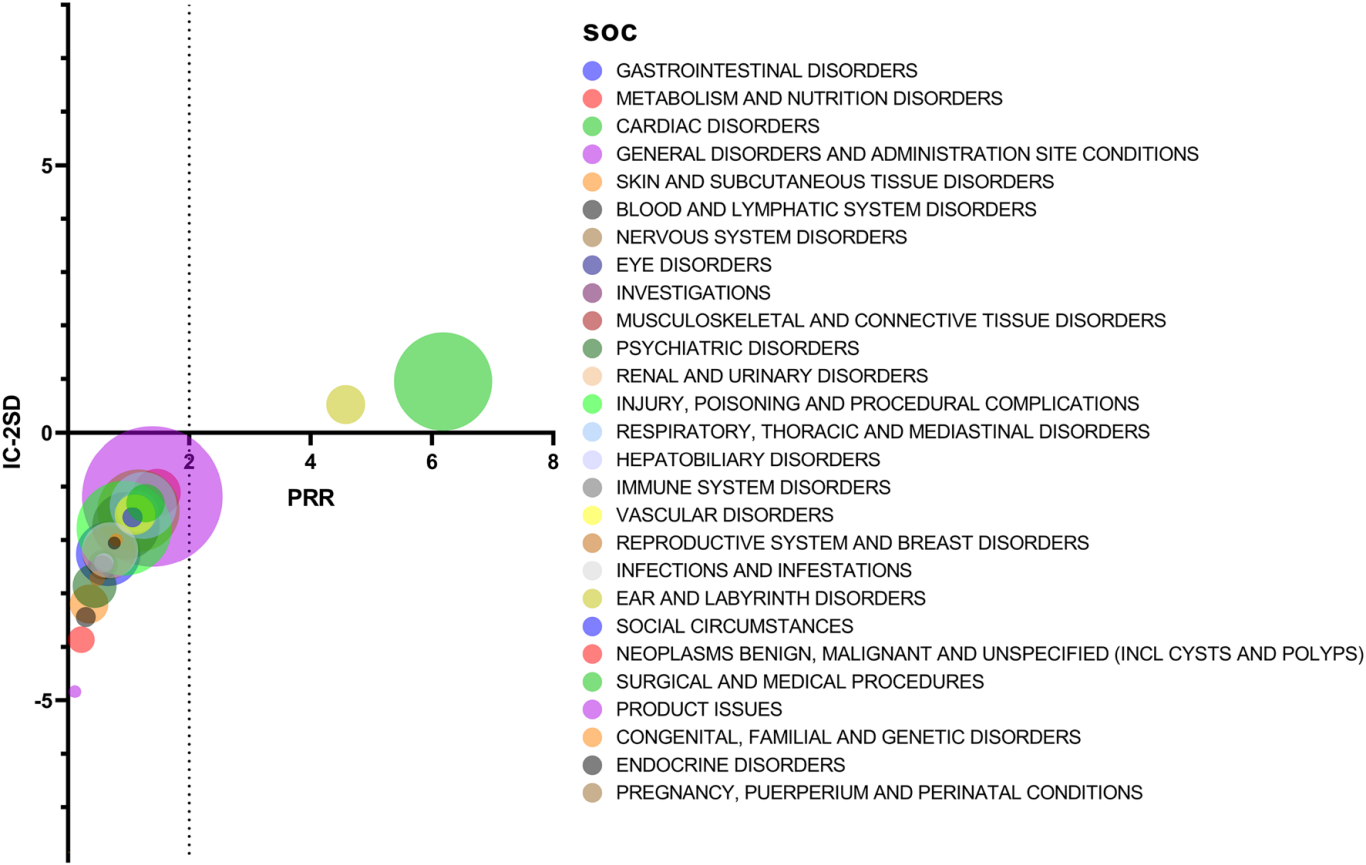
Significance analysis of SOCs.

Adverse events related to tafamidis involved 27 SOCs, cardiac disorders (a = 2549), and ear and labyrinth disorders (a = 398) were screened as positive signals. The result is shown in Fig. 3.

#### Positive AE signals under positive SOC

**Figure 4:**
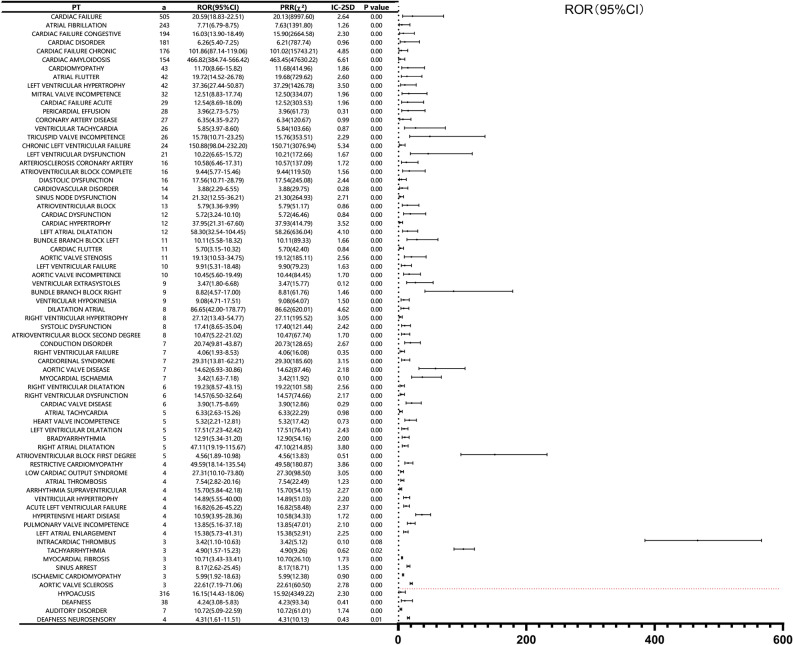
Positive signals under positive SOCs.

Screening all the adverse event signals in the two positive SOCs of cardiac disorders and ear and labyrinth disorders, we obtained a total of 71 positive adverse event signals, cardiac disorders (a = 67), the ear and labyrinth disorders (a = 4), as shown in Fig. 4. The highest number of reported cases in the Cardiac disorders system was cardiac failure [(a = 505, ROR 20.59 (18.83–22.51)], the most significant signal was cardiac amyloidosis [(a = 154, ROR 466.82 (384.74–566.42)]. Hypoacusis [(a = 316, ROR 16.15(14.43–18.06)] is the highest and the most significant AE signal in ear and labyrinth disorders. Except for intracardiac thrombus, there was a significant difference between the frequency of tafamidis appearing as a positive AE signal in a positive SOC and the frequency of the same signals for the other drugs in the database (P < 0.05).

### Results of positive signal mining in subgroup

#### Positive SOC signals in subgroup

**Figure 5:**
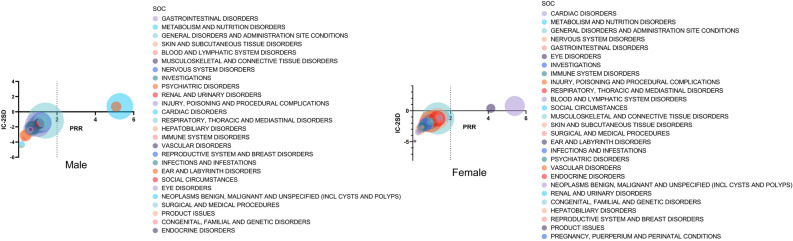
Positive SOC signals in subgroup.

We obtained reports from 5984 males and 1460 females in all AE reports. Detection of SOC signals by gender as shown in Fig. 5. In both males and females, ear and labyrinth disorders and cardiac disorders were screened to be positive signals.

#### Top 20 positive AE signals in subgroup

**Figure 6:**
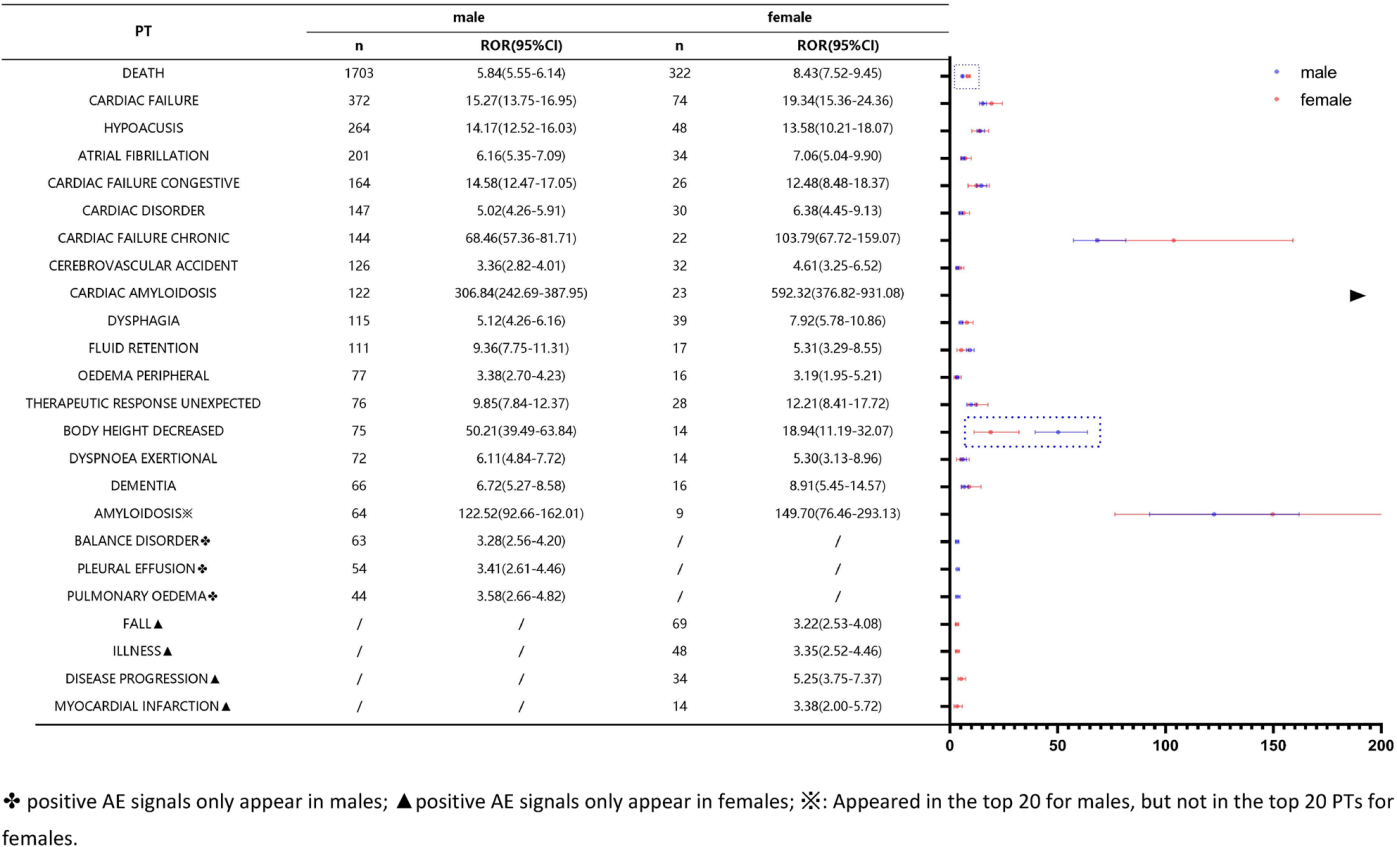
Top 20 positive AE signals in subgroup.

A comparison of the top 20 positive AE signals in the gender subgroups is shown in Fig. 6. Fall, illness, disease progression, and myocardial infarction were unique to females, and balance disorder, pleural effusion, and pulmonary oedema were unique to males. Adverse events such as death, hypoacusis, cardiac failure chronic, and so on were reported positively in different gender subgroups. Body height decreased and death 95%CI did not overlap and significant differences existed. The death signal strength was significantly higher in females than in males, and the body height decreased signal strong enemy was significantly higher in males than in females.

#### Potential AE signals

Compared the AEs mentioned in the clinical study of tafamidis with the AE signals obtained from this data mining, urinary tract infection bacterial [(*a* = 5, ROR 4.60 (1.91–11.07)] screened to show positive AE signals, see Table 2. In the gender subgroups, only the male group had a positive AE signal for urinary tract infection bacterial screening [(*a* = 4, ROR 7.07 (2.64–19.01)].

**Table 2:** Potential AE signals.

PT	a	ROR (95% CI)	PRR (χ^2^)	IC-2SD	Male				Female			
					a	ROR (95% CI)	PRR (χ^2^)	IC-2SD	a	ROR (95% CI)	PRR (χ^2^)	IC-2SD
Urinary tract infection bacterial*	5	4.60 (1.91–11.07)	4.59 (13.99)	0.53	4	7.07 (2.63–19.01)	7.07 (20.50)	1.12	1	4.59 (0.65–32.64)	4.59 (2.80)	0.53
Hypertension	64	0.93 (0.73–1.19)	0.93 (0.35)	− 1.77	44	0.87 (0.65–1.17)	0.87 (0.81)	− 1.86	20	1.48 (0.95–2.29)	1.47 (3.06)	− 1.11
Hypotension	76	1.20 (0.96–1.51)	1.20 (2.57)	− 1.40	64	1.11 (0.87–1.42)	1.11 (0.68)	− 1.52	11	0.99 (0.55–1.79)	0.99 (0.00)	− 1.68
Hypoaesthesia	71	1.59 (1.26–2.00)	1.58 (15.31)	− 1.00	50	1.79 (1.36–2.36)	1.79 (17.30)	− 0.83	19	1.90 (1.21–2.98)	1.89 (8.02)	− 0.75
Platelet count decreased	15	0.39 (0.23–0.64)	0.39 (14.63)	− 3.04	13	0.39 (0.23–0.67)	0.39 (12.42)	− 3.02	1	0.15 (0.02–1.07)	0.15 (4.76)	− 4.39
Pneumonia	121	1.15 (0.96–1.37)	1.15 (2.31)	− 1.47	92	1.01 (0.83–1.24)	1.01 (0.02)	− 1.65	22	1.17 (0.77–1.78)	1.17 (0.53)	− 1.44
Constipation	63	0.87 (0.68–1.12)	0.87 (1.15)	− 1.86	52	0.99 (0.75–1.30)	0.99 (0.01)	− 1.68	10	0.70 (0.37–1.30)	0.70 (1.31)	− 2.19
Hepatic function abnormal	10	0.83 (0.45–1.55)	0.83 (0.33)	− 1.93	10	0.78 (0.42–1.46)	0.78 (0.59)	− 2.02	–			
Diarrhoea	151	0.67 (0.58–0.79)	0.68 (23.45)	− 2.23	102	0.62 (0.51–0.76)	0.62 (23.32)	− 2.35	38	0.83 (0.61–1.15)	0.84 (1.25)	− 1.93
Haematuria	13	1.31 (0.76–2.26)	1.31 (0.95)	− 1.28	12	0.94 (0.53–1.65)	0.94 (0.05)	− 1.76	1	0.92 (0.13–6.51)	0.92 (0.01)	− 1.79
Gastrointestinal haemorrhage	17	0.83 (0.51–1.33)	0.83 (0.61)	− 1.94	15	0.68 (0.41–1.13)	0.68 (2.22)	− 2.22	1	0.33 (0.05–2.32)	0.33 (1.38)	− 3.28
Renal failure	85	1.94 (1.57–2.41)	1.94 (38.76)	− 0.71	67	1.32 (1.04–1.68)	1.32 (5.23)	− 1.27	10	1.67 (0.90–3.11)	1.67 (2.69)	− 0.93

*Positive signal.

### AE induce time analysis

**Figure 7:**
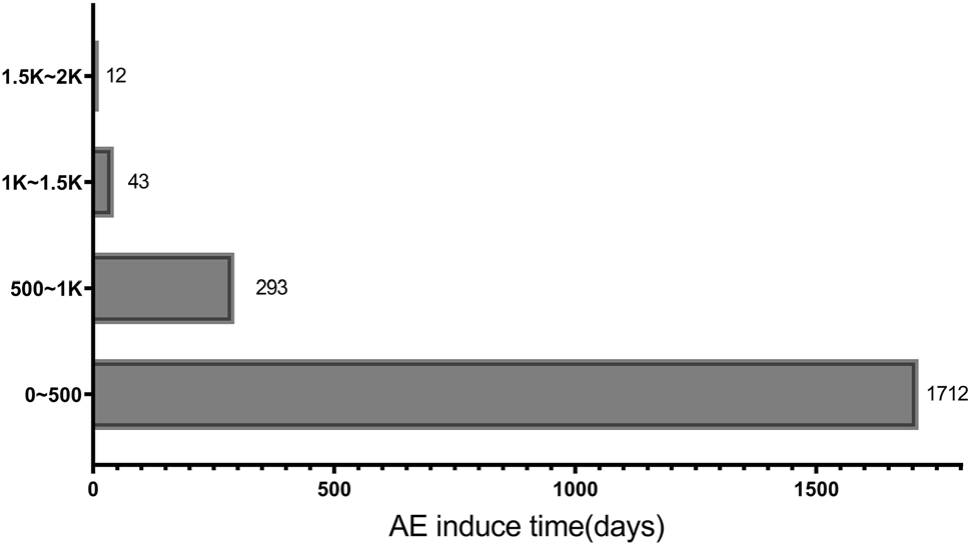
AE induce time of all reports.

**Figure 8:**
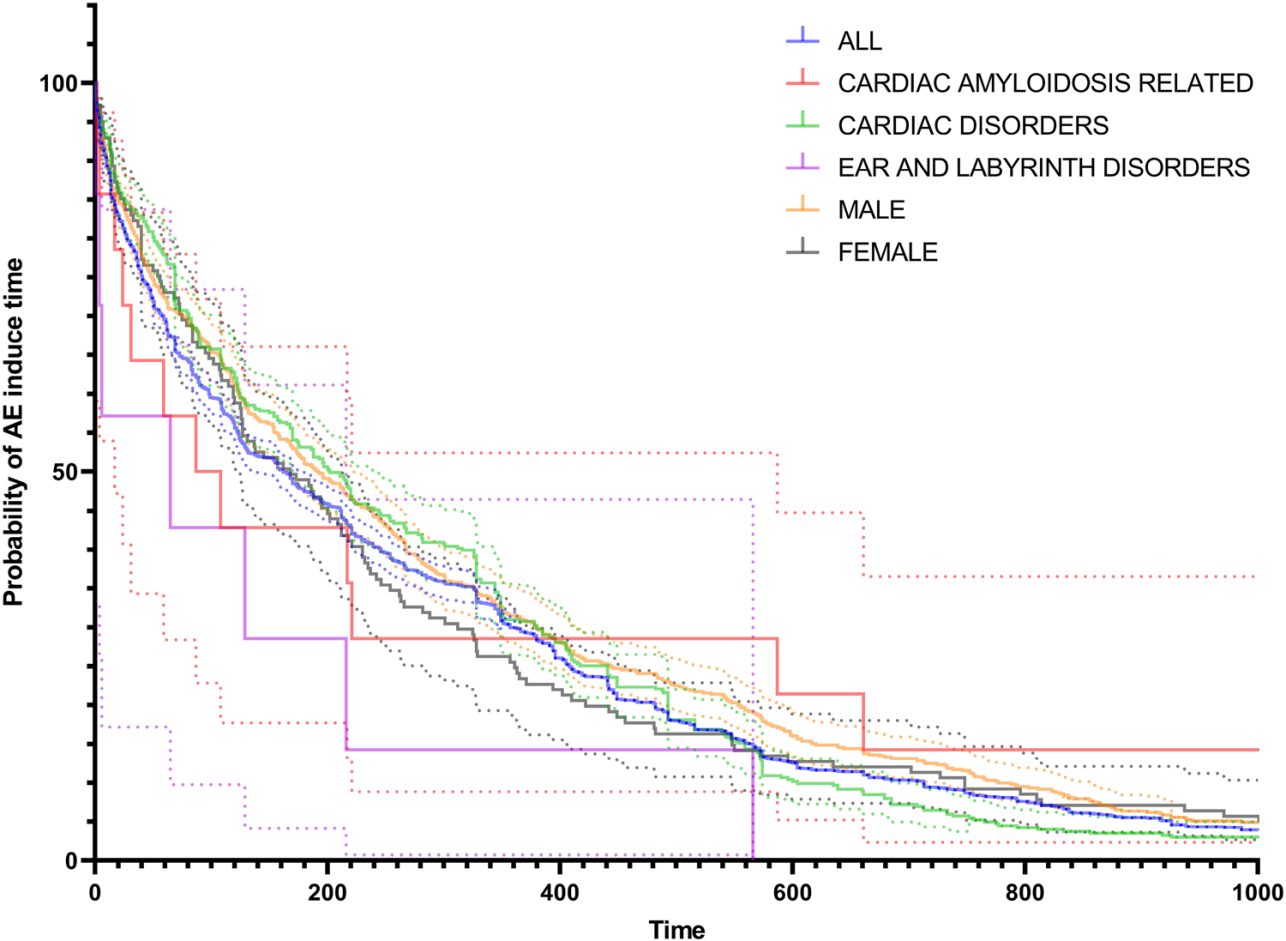
AE induce time survival analysis.

We screened a total of 2060 eligible AE reports in all AE reports and analyzed the percentage ratio (Figs. 7 and 8). Adverse drug events are reported throughout the treatment period. The highest number of reports of AE was reported after 0–500 days of medication, accounting for 83.11% of all reports. Cardiac amyloidosis related, cardiac disorders, ear and labyrinth disorders, males, and females showed no significant differences in the induce time survival analysis and nonparametric Kruskal Wallis test (*P* = 0.076).

## Discussion

Tafamidis, as the world's first and only approved oral drug for the treatment of ATTR-CM, in clinical trials and literature reviews, with good efficacy and tolerability and independent of age subgroup. The tafamidis package insert and some clinical studies concluded that the adverse events of tafamidis were not significantly different from the placebo group^5,15,16^. However, some clinical studies have found the presence of urinary tract infection bacterial, hypertension, hypoaesthesia, and platelet count decreased adverse events after clinical application of tafamidis^12–14^. This study mined and analyzed all post-marketing AE reports for tafamidis based on the FAERS database, compared with previous clinical studies and was dedicated to discovering the potential adverse reaction risks of tafamidis.

With tafamidis being marketed in many countries around the world, reports of adverse reactions in FAERS with tafamidis as the first suspected drug have increased each year. Our study counted baseline information on patients, the vast majority of tafamidis users were males (73.24%) and over 65 years of age (59.30%), consistent with the epidemiologic findings of patients in the TTR study^3^. Following tafamidis treatment, 28.46% of patients reported death outcome. In the comparison of signal intensities, the signal intensity of death was significantly higher in females than in males, which may be related to the fact that females have a later onset of disease, which can be easily missed, and that males have a better prognosis after treatment^3^. We found that although adverse events for tafamidis were reported throughout the treatment period, the longer the treatment time, the fewer adverse events were reported.

In our study, we found a large number of positive SOC signals and positive AE signals associated with primary diseases, such as cardiac disorders and cardiac amyloidosis. When the TTR protein misfolds, it can form amyloid fibrils that deposit in the heart causing heart failure, heart conduction block, or arrhythmia such as atrial fibrillation^1,17,18^. The large number of positive AE signals such as cardiac failure, atrial fibrillation, and cardiomyopathy that were found in this study are consistent with the disease presentation of ATTRamyloidosis. We believe that ATTR is a rare disease that is not well understood, so there can be instances where ATTR-related symptoms are reported as adverse drug reactions. Consumers and Medical Doctors accounted 75.15% of the tafamidis AE report recorder statistics, and this research data confirms our view. Disease progression in ATTR is insidious, often lasting years or even decades, and manifests as symptoms such as dyspnea, fatigue, peripheral edema, and palpitations. Tafamidis slowed the progression of ATTR amyloidosis, but the above manifestations may still occur^19,20^. Clinicians and patients using tafamidis should be sufficiently aware of the manifestations of ATTR disease progression and distinguish them from adverse drug reactions.

Currently, there are more than 18,100 drugs that have been shown to be ototoxicity, and 35.2% of acquired deafness is related to drug use; however, the mechanism of action behind ototoxicity is not yet fully understood. This data mining obtained ear and labyrinth disorders positive SOC signal and hypoacusis, auditory disorder, deafness, deafness neurosensory positive AE signals. The results suggest that tafamidis has the potential risk of inducing adverse events in ear and labyrinth disorders. This prominence of SOC/AE positive signal may be associated with extracardiac manifestations caused by mutations in a single nucleotide of the patient's TTR gene, also possible that the clinical studies didn't cover enough cases to observe the corresponding adverse effects of tafamidis^17^. Some drugs can cause damage to the structural function of the ear by themselves or by their toxic metabolites, for example, Lidocaine can have a toxic effect of transmitting directly into the inner ear through the round window membrane. Some drugs such as furosemide can reduce blood flow to the side wall of the cochlea and cause ischemia and hypoxia in the stria vascularis to affect hearing^21^. Based on the serious impact of adverse effects in hearing impairment on patients' life quality, the cause of tafamidis-induced high SOC expression requires more intensive clinical observation. Whether the stabilization of TTR proteins by tafamidis affects the biochemical disruption of cell membranes, leading to an impact on ear auditory cell metabolism remains to be clinically investigated.

Previous studies in the clinical application of tafamidis found that the incidence of urinary adverse reactions was higher in the treatment group compared with the placebo group^15^. In our study, urinary tract infection bacterial was tested to be a positive AE signal, which also suggests that there is a potential risk of adverse reactions. In addition, this adverse reaction may be more frequent in the male subgroup. Drug induced changes in urine output and urethral pH may lead urinary tract infection bacterial adverse reactions. In addition, patients with ATTR have a poor cardiac function, and progress of the disease to the end stage of heart failure will severely limit the intake, which will result to a low daily urine output. This is also a possible reason for the strong expression of urinary tract infection bacterial AE positive signal. Based on this is recommended that patients should drink appropriate amounts of water and pay extra attention to urinary tract care to prevent bacterial infections during treatment with tafamidis. The effects of tafamidis on the urinary system deserve further clinical investigation, as well as exploration of strategies to minimize urinary effects in clinical practice.

In general, adverse event data mining can complement the deficiencies of pre-marketing clinical studies of drugs and detect late or rare adverse drug events. However, positive signals may have some bias or ommitted due to the influence of patients' basic diseases, the number of target drugs and the number of target adverse reactions. This is the most common limitation of adverse event data mining studies^6^. For example, previous clinical studies mentioned that tafamidis may cause hypotension, inadequate anesthesia, diarrhoea and decreased platelet counts, but our data study did not result these associated positive AEs. We extracted 151 cases of diarrhea reported after tafamidis administration, which is a relatively high number of reported adverse reactions for tafamidis. However, since diarrhea is the most common adverse reaction to pharmaceuticals, the huge number of reported adverse reactions to diarrhea for drugs other than tafamidis would show up in the signal calculation as a high denominator value, which results ultimately in a low signal value, leading to diarrhea not satisfying the inclusion criteria for a positive signal and being screened out. To reduce this interference or omission of reported valid adverse event signals, and to reduce the number of false-positive and false-negative signals, the BCPNN and MHRA methods, which have better stability and the sensitivity of the results, were jointly used in this study. The results of this adverse reaction data mining can be used to inform clinical safety studies of tafamidis, but the consistency of the final results of the screening with the actual clinical manifestations still needs to be further evaluated. In addition, Tafamidis is a new drug marketed in 2019 for rare disease treatment with expensive price^22,23^, and the narrow audience and short application time may lead to a large number of adverse events reported about tafamidis as not being accessible to the database. Based on this, it is recommended that tafamidis be appropriately priced downward so that patients with ATTR-CM, especially those with more difficult economic conditions, can afford the cost of treatment. Improving the quality of patient survival and helping people to understand this new drug more comprehensively at the same time. At last, FDA adverse event reporting is not required to demonstrate the causal relationship between the adverse event and the medicine, and disease-related positive signals are likely to perform as false positives. We recommended that the assessment of causality between a drug and adverse events should be added to the process of reporting FAERS, thus greatly increasing the credibility of safety vigilance studies of post-marketing drugs.

## Conclusion

Our study provides information on real-world-based post-marketing pharmacovigilance of tafamidis, a large amount of data mining work suggests that tafamidis shows a favorable safety profile. We found that tafamidis has a potential adverse event risk of inducing hearing system damage and urinary tract infections, and no signals of potentially fatal or disabling adverse reactions were identified. Tafamidis needs to be taken for a long period of time and is expensive. It is recommended that extra attention be paid to the safety of the drug along with the efficacy studies of tafamidis, and it is recommended that clinical studies be conducted in a wider group of patients to assess the long-term safety of tafamidis.

### Supplementary Information

Supplementary Information.

## Data Availability

All data is publicly available on the FDA website (https://fis.fda.gov/extensions/FPD-QDE-FAERS/FPD-QDE-FAERS.html).
