# Supplementary material for: Analysis of post-market adverse events of tafamidis base on the FDA adverse event reporting system
Source: Sci Rep. 2024 Jun 13;14:13691. doi: 10.1038/s41598-024-64697-y (PMC11176370; doi:10.1038/s41598-024-64697-y)
Supplement: Supplementary file 1 — Supplementary Information. [file 41598_2024_64697_MOESM1_ESM.docx]

# Specific Formulas and Analytical Methods

| ITEM | TARGET AEs | OTHER AEs | SUMs |
| --- | --- | --- | --- |
| TARGET DRUG | a | b | a + b |
| OTHER DRUGs | c | d | c + d |
| SUMs | a + c | b + d | a + b + c + d |

| ALGORITHMS | EQUATION |
| --- | --- |
| BCPNN | $\gamma_{\mathrm{ij}}1,\alpha_{i}=\beta_{j}=1,\alpha=\beta=1,c_{\mathrm{ij}}=a,c_{i}=a+b,$  $c_{j}=a+b,c_{j}=a+c,N=a+b+c+d$ |
|  | $\gamma=\gamma_{\mathrm{ij}}\frac{(N+\alpha)(N+\beta)}{(c_{i}+\alpha_{i})(c_{j}+\beta_{j})}$ |
|  | $E(\mathrm{IC}_{\mathrm{ij}})=\log_{2} \frac{(c_{\mathrm{ij}}+\gamma_{\mathrm{ij}})(N+\alpha)(N+\beta)}{(N+\gamma)(c_{i}+\alpha_{i})(c_{j}+\beta_{j})}=\log_{2} \frac{{(c}_{\mathrm{ij}}+\gamma_{\mathrm{ij}})\gamma}{(N+\gamma)}$ |
|  | $V(\mathrm{IC}_{\mathrm{ij}})=\frac{\frac{N-c_{\mathrm{ij}}+\gamma-\gamma_{\mathrm{ij}}}{{(c}_{\mathrm{ij}}+\gamma_{\mathrm{ij}})(1+N+\gamma)}+\frac{N-c_{i}+\alpha-\alpha_{i}}{(c_{i}+\alpha_{i})(1+N+\alpha)}+\frac{N-c_{j}+\beta-\beta_{i}}{(c_{i}+\beta_{j})(1+N+\beta)}}{{(log 2)}^{2}}$ |
| CRITERIA | IC-2SD >0, (IC=E (IC_ij_), SD=$\sqrt{V({IC}_{ij})}$ ) |
| MHRA | $PRR=\frac{\left[ a\times\left( a+b \right) \right]}{\left[ c\times\left( c+d \right) \right]}$ |
|  | $\chi^{2}=\frac{\left( \left\vert a\times d-b\times c \right\vert-\frac{n}{2} \right)^{2}\times n}{\left( a+b \right)\left( a+c \right)\left( c+d \right)\left( b+d \right)}$ |
| CRITERIA | PRR ≥ 2, χ^2^ ≥ 4, and a ≥ 3 |

Note: *a* means the number of reports containing both the target drug and target AE/SOC; *b* means number of reports containing other AE/SOC of the target drug; *c* means the number of reports containing the target AE/SOC of other drugs; *d* means the number of reports containing other drugs and other AE/SOC.

When the calculation result is satisfied with both a≥3, χ2≥4, proportional reporting ratio (PRR)≥2, lower limit of the information component (IC-2SD) >0, one positive signal is recognized.
